# Combinatorial G-CSF/AMD3100 Treatment in Cardiac Repair after Myocardial Infarction

**DOI:** 10.1371/journal.pone.0104644

**Published:** 2014-08-14

**Authors:** Constantin Rüder, Tobias Haase, Annalena Krost, Nicole Langwieser, Jan Peter, Stefanie Kamann, Dietlind Zohlnhöfer

**Affiliations:** 1 Berlin Brandenburg Center for Regenerative Therapies (BCRT), Berlin, Germany; 2 Department of Cardiology, Campus Virchow Klinikum, Charité Berlin, Germany; Leiden University Medical Center, Netherlands

## Abstract

**Aims:**

Several studies suggest that circulating bone marrow derived stem cells promote the regeneration of ischemic tissues. For hematopoietic stem cell transplantation combinatorial granulocyte-colony stimulating factor (G-CSF)/Plerixafor (AMD3100) administration was shown to enhance mobilization of bone marrow derived stem cells compared to G-CSF monotherapy. Here we tested the hypothesis whether combinatorial G-CSF/AMD3100 therapy has beneficial effects in cardiac recovery in a mouse model of myocardial infarction.

**Methods:**

We analyzed the effect of single G-CSF (250 µg/kg/day) and combinatorial G-CSF/AMD3100 (100 µg/kg/day) treatment on cardiac morphology, vascularization, and hemodynamics 28 days after permanent ligation of the left anterior descending artery (LAD). G-CSF treatment started directly after induction of myocardial infarction (MI) for 3 consecutive days followed by a single AMD3100 application on day three after MI in the G-CSF/AMD3100 group. Cell mobilization was assessed by flow cytometry of blood samples drawn from tail vein on day 0, 7, and 14.

**Results:**

Peripheral blood analysis 7 days after MI showed enhanced mobilization of white blood cells (WBC) and endothelial progenitor cells (EPC) upon G-CSF and combinatorial G-CSF/AMD3100 treatment. However, single or combinatorial treatment showed no improvement in survival, left ventricular function, and infarction size compared to the saline treated control group 28 days after MI. Furthermore, no differences in histology and vascularization of infarcted hearts could be observed.

**Conclusion:**

Although the implemented treatment regimen caused no adverse effects, our data show that combinatorial G-CSF/AMD therapy does not promote myocardial regeneration after permanent LAD occlusion.

## Introduction

Cytokine mediated mobilization of peripheral blood stem cells for autologous stem cell transplantation is a generally accepted therapeutic option for the hematopoietic reconstitution after myoablative chemotherapy. The clinically used cytokine granulocyte-colony stimulating factor (G-CSF) is known to mobilize various subsets of hematopoietic stem and progenitor cells (HSPC) into blood circulation that may contribute to tissue repair. Additionally G-CSF was shown to have anti-apoptotic, anti-inflammatory and antioxidant effects [1], [2], [3]. These findings raised expectations of G-CSF as a promising therapeutic avenue in tissue regeneration.

Especially in the field of ischemic heart disease numerous studies investigated the efficacy of G-CSF induced stem cell mobilization in myocardial regeneration. While early animal studies and small clinical trials indicated beneficial effects on cardiac regeneration, these results were later challenged by studies that could not confirm these positive effects or even reported deleterious effects of G-CSF therapy on cardiac recovery (for review see [4], [5], [6]). The missing benefit of G-CSF induced mobilization of progenitor cells might be due to a reduced homing capacity as G-CSF treatment results in significant downregulation of important adhesion molecules on mobilized cells [7].

Besides G-CSF, the CXCR4 antagonist AMD3100 (AMD) was shown to rapidly mobilize stem cells by reversibly disrupting the interaction between CXCR4 and SDF-1α that tethers stem cells to the bone marrow (BM) environment [8]. In patients that do not respond to single G-CSF treatment a combination of G-CSF and AMD has shown to effectively mobilize hematopoietic stem cells (HSC) from the BM [9]. Moreover combinatorial G-CSF/AMD therapy was shown to be superior to single G-CSF therapy with respect to HSC numbers and is clinically approved for autologous HSC mobilization [10]. Preclinical studies on AMD in tissue regeneration showed that acute application leads to enhanced vascularization of ischemic tissues [11], [12] while continuous AMD treatment has deleterious effects on tissue regeneration [13], [14]. This effect was attributed to the crucial role of the CXCR4/SDF-1α axis in stem cell homing towards injured tissues [7], [15].

On the basis of these results we explored possible beneficial effects of combinatorial G-CSF/AMD therapy in myocardial regeneration in a mouse model of MI. We applied a treatment regimen were G-CSF administration started directly after induction of MI for 3 consecutive days followed by a single dose of AMD in order to attain positive effects on stem cell mobilization while avoiding negative effects on stem cell homing.

## Methods

### Surgical induction of myocardial infarction and study design

Eight to ten weeks old male FVB/NJ mice (Charles River) were anaesthetized with an intraperitoneal injection of midazolam (5.0 mg/kg), fentanyl (0.05 mg/kg), and medetomidin (0.5 mg/kg). The animals were intubated and ventilated using a rodent ventilator (MiniVent, Hugo Sachs) with a stroke volume of 0.2 ml and respiration rate of 200 strokes/min. Inhalation anesthesia was maintained with 1.5% isoflurane through a vaporizer with 100% oxygen. After left lateral thoracotomy at the left third intercostal space, the left anterior descending coronary (LAD) was ligated with 7–0 prolene sutures (Ethicon) 1 mm below the tip of the left atrial auricle. The chest and skin were closed with 6–0 vicryl (Ethicon) sutures. The sham group underwent the same procedure except for the ligation of the LAD. After induction of MI animals were randomly divided into 3 groups and stem cell mobilization was induced by the following dosing regimen:

1) G-CSF (250 µg/kg/day; Amgen GmbH) subcutaneously (s.c.) starting 1 h post-MI and then daily on days 1 and 2; 2) Combination of G-CSF s.c. starting 1 h post-MI and then daily on days 1 and 2+ AMD3100 (5 mg/kg/day; Sigma-Aldrich) s.c. as single dose on day 3 post-MI; 3) the control MI group received at the same time points equal volumes of saline (0.9% NaCl). Data acquired from control MI and sham groups contributed in parallel to another study but were pooled with new animals [16]. Postoperative, mice were housed singly in enriched standard cages with free access to food and water. Mice were monitored three times per day during the first three days and two times per day from day 4–7. After this acute phase, mice were monitored one time per day. During the first 7 days after MI analgesia was maintained by buprenorphine application (0.1 mg/kg). The state of health of mice was recorded on a score sheet. Animals that died in this study after induction of MI deceased due to acute heart failure or heart rupture as a result of the intervention. Euthanasia on the basis of humane endpoints was not done, but humane endpoints were included in the applied animal care guidelines approved by the local authorities Landesamt für Gesundheit und Soziales (LAGeSo) and Gesellschaft für Versuchstierkunde (GV-SOLAS). Humane endpoints were: automutilation, sepsis, local infection, dyspnea/apnea, apathy, dehydration, weight loss over 20% and drastic worsening of the general health condition on the basis of the score sheet rating. After 28 days mice were sacrificed in deep isoflurane anesthesia by cervical dislocation by trained personnel. All animal procedures were performed in accordance with institutional and federal animal care guidelines and approved by the ethics committee of the LAGeSo (Permit Number: G003109).

### Flow cytometry analysis of peripheral blood

Whole blood samples were drawn from the tail vein 0, 7 and 14 days after MI and circulating white blood cells (WBC) were counted with an animal blood counter (Scil Vet abc). For fluorescence activated cell sorting (FACS) analysis, blood mononuclear cells were separated via gradient-density centrifugation using Histopaque-1083 (Sigma-Aldrich). Cells were blocked with normal rat serum and anti-CD16/32 monoclonal antibody (mAb) (clone 93) in FACS buffer (PBS, 0.5% BSA, 0.05% NaN_3_) and incubated with Alexa Fluor 647-labeled anti-Flk-1/VEGFR2 (clone 89B3A5), Phycoerythrin (PE)-conjugated anti-Ly-6A/E (Sca-1) (clone D7), and allophycocyanin(APC)-labeled anti-CD117 (c-kit) (clone 2B8) (all purchased from Biolegend). Appropriate isotype controls were always included. Cells were analyzed on FACSCanto II flow cytometer using FACSDiva software (BD Biosciences) and analyzed with FlowJo software (TreeStar).

### Hemodynamic measurements

Evaluation of ventricular pressure–volume relationships was done 28 days after surgical induction of MI in isoflurane anesthetized ventilated mice as described above. A 1.4F polyimide pressure-conductance catheter (Millar Instruments) was inserted through the right carotid artery into the left ventricle to record baseline pressure-volume loops in the closed chest. Conversion of raw conductance data to calibrated volumes was performed by determination of parallel conductance (Vp) using hypertonic saline dilution method [17], [18]. Afterward, mice were euthanized in deep anesthesia and hearts were excised. Measurements and data analysis were performed by a blinded person using LabChart® Software (AD Instruments).

### Histology and Immunofluorescence

At day 28, hearts were excised, fixed overnight with 4% formalin/PBS-buffered and embedded in paraffin. Transversal sections of a thickness of 3 µm were cut from apex to base and mounted on glass slides for histological and immunhistochemical staining. Masson trichrome (MT) staining was done according to standard protocols. Infarction size was determined using midline length measurement on MT stained sections from the surgical LAD occlusion to the base [19]. For the quantification of fibrosis, blue stained areas of sequential MT stained heart sections were determined and correlated to the whole heart section area using ImageJ software. Additionally, infarction size was determined by staining with 2% tri-phenyltetrazolium chloride (TTC). Therefore, hearts were frozen at −20°C and cut in semi frozen state into five equally thick sections. Slices were then incubated in TTC solution for 15 min at 37°C and fixed in 10% formalin. Viable myocardium stained red while the infarcted area appeared pale-white. The area of infarction was measured in each slice with ImageJ (1.44; National Institutes of Health) software and expressed as percentage of the entire left ventricular area (including septum). Vascularization was evaluated by immunostaining with CD31/PECAM-1 (sc-1506-R M-20 clone, Santa Cruz Biotechnology) and α-smooth muscle actin (clone 1A4, Sigma-Aldrich) primary antibody followed by incubation with respective AlexaFluor-labeled secondary antibodies (Invitrogen). Images were acquired with a Zeiss Axioskop microscope. Capillary density was determined by counting CD31/PECAM-1 positive vascular structures in three randomly chosen high-power fields (each 40000 µm^2^) in 10 sections per heart (n = 5–8 for each group) within the border zone, infarcted and remote area. Alpha-smooth muscle actin positive vascular structures were counted per area assessed with ImageJ software. Both parameters are expressed as positive stained vascular structures per mm^2^.

### Statistics

Data are reported as mean value ± standard error of the mean (SEM), and were analyzed by two-tailed unpaired Student's t-test. A p-value of less than 0.1 was considered as a trend a p-value less than 0.05 was considered significant. Group comparisons were performed using One-way ANOVA followed by the Tukey's test. Survival analysis was assessed by Kaplan-Meier method.

## Results

### Mobilized peripheral blood cells

**Figure 1:**
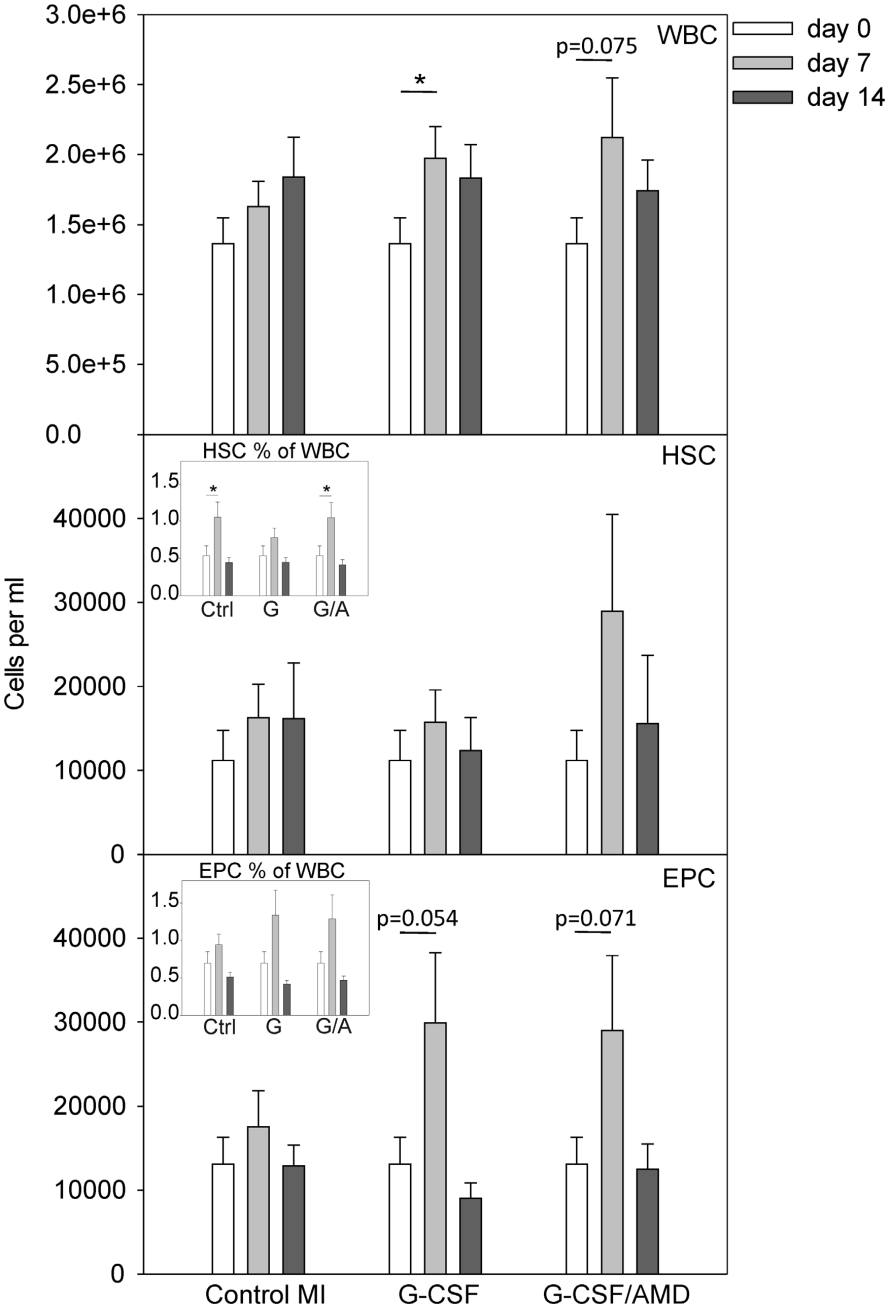
Mobilized peripheral blood cells after MI. Circulating white blood cells were counted before (day 0), 7 and 14 days after MI. **(upper panel)** G-CSF and combinatorial G-CSF/AMD treatment enhances white blood cell numbers 7 days after MI (*p<0.05 G-CSF day 7 vs. day 0, p<0.075 G-CSF/AMD day 7 vs. day 0). Flow cytometry analysis on peripheral blood mononuclear cells was done before (day 0) and 7 and 14 days after induction of MI in control MI, G-CSF and G-CSF/AMD treated mice. **(middle panel)** The absolute numbers of circulating c-Kit^+^Sca-1^+^ double positive HPC were not different between control MI and drug treated groups. The HSC fraction was significantly increased in the control and G-CSF/AMD group (*p<0.05 vs. day 0), but not in G-CSF treated mice **(middle panel inset)**. **(lower panel)** Flk-1^+^Sca-1^+^ double positive EPC mobilization peaked 7 days after MI in drug treated mice (p<0.054 G-CSF day 7 vs. day 0; p<0.071 G-CSF/AMD day 7 vs. day 0). EPC fractions were increased upon drug treatment, but did not reach statistical significance **(lower panel inset)**. Data represent means ± SEM. (n>10 per group).*p<0.05.

Circulating white blood cells (WBC) and progenitor cells were determined before (0 days) and after MI in drug treated mice and untreated controls. Treatment with G-CSF (*p<0.05 vs. day 0) and G-CSF/AMD (p = 0.075 vs. day 0) enhanced the MI induced mobilization of circulating WBC, without reaching statistical significance compared to the untreated group 7 days after MI (Figure 1 upper panel). Mobilization of HSC and endothelial progenitor cells (EPC) was analyzed by co-expression of receptor tyrosin kinase c-kit and stem cell antigen-1 (Sca-1) or fetal-liver kinase-1 (Flk-1) on peripheral blood mononuclear cells, respectively (Figure S1). Although not statistically significant, G-CSF/AMD3100 treatment led to elevated absolute numbers of c-Kit/Sca-1 positive HSC into circulation compared to single G-CSF administration and untreated control mice 7 days after MI (Figure 1 middle panel). The percentage of HSC was significantly increased in the control and G-CSF/AMD group (*p<0.05 vs. day 0), but not in G-CSF treated mice (middle panel inset). Absolute numbers of EPC were elevated by single treatment with G-CSF (p = 0.054 vs. day 0) and combinatorial G-CSF/AMD treatment (p = 0.071 vs. day 0) 7 days after MI (Figure 1 lower panel). Furthermore, EPC percentages were increased upon drug treatment, but did not reach statistical significance (lower panel inset). No synergistic augmentation of circulating cell numbers could be observed when G-CSF treatment was combined with AMD after MI.

### Survival

**Figure 2:**
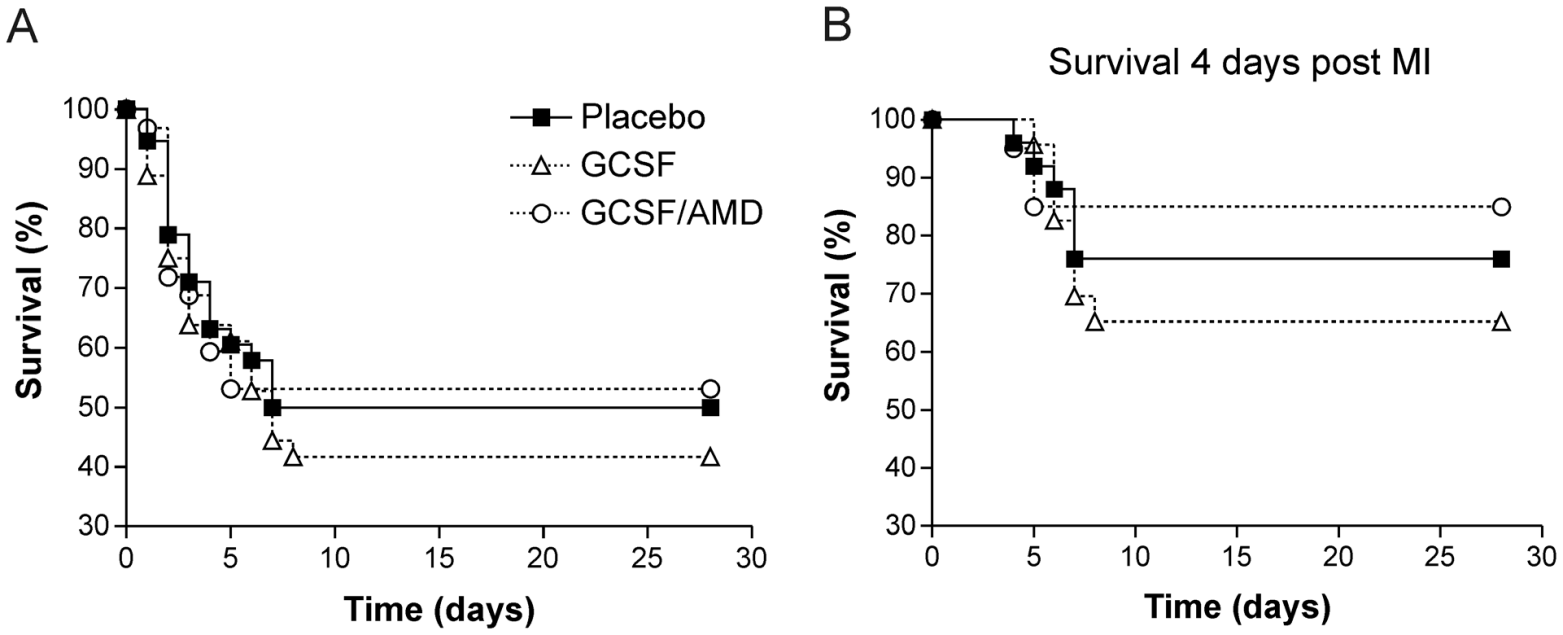
Cumulative Kaplan-Meier survival analysis. Kaplan-Meier survival curve of control MI and drug-treated mice during the observation period of 28 days after MI. Treatment of mice with G-CSF or G-CSF/AMD did not improve the (**A**) overall survival and did not alter (**B**) the mortality of mice that survived the first 4 days after MI.

A total of 106 mice (38 control MI, 36 G-CSF, 32 G-CSF/AMD) were included into cumulative Kaplan-Meier survival analysis. There were no statistical differences in overall mortality between saline and drug treated groups (Figure 2 A). In order to prevent that early deaths after surgery has masked beneficial effects, the 70 (27 control MI, 23 G-CSF, 20 G-CSF/AMD) animals that survived for the first 4 days after MI were included in a modified Kaplan-Meier survival analysis. However, even after exclusion of mice that died early after MI there were no significant differences in survival rates between saline and drug treated animals (Figure 2 B). Post-mortem examination confirmed that all dead mice suffered from MI.

### Heart function and infarction size

Surgical induction of MI severely impaired cardiac function compared to sham-operated animals as assessed by hemodynamic measurements 28 days after surgery. In sham-operated mice the left ventricular ejection fraction (EF) was 66.2%±5.2 and declined to 23.8%±2.9 (p<0.001) in control MI mice. G-CSF and G-CSF/AMD treatment did not improve EF 28 days after MI (Table 1). Detailed examination of P-V loop derived hemodynamic parameters confirmed a severely decreased cardiac function in all MI groups (see Table 1). The parameters stroke work (SW), stroke volume (SV), cardiac output (CO) and the rate in fall of ventricular pressure (dP/dt min) were significantly reduced, while end-systolic and end-diastolic volumes (Ves, Ved) increased in control MI compared to sham-operated mice. Drug treated animals showed significantly reduced EF, SW, SV and CO compared to sham-operated animals. The rates in rise and fall of ventricular pressure (dP/dt max, dP/dt min) and Ves, Ved were not significantly altered in drug treated compared to sham-operated mice. However, in comparison to control MI animals, drug treatment did not significantly improve any of the recorded parameters of left ventricular function (see Table 1).

**Table 1:** Left ventricular hemodynamics recorded by pressure-volume catheterization in the closed chest 28 days after LAD ligation.

	Sham (n = 11)	Control MI (n = 11)	GCSF (n = 5)	GCSF/AMD (n = 9)
HR (bpm)	443.9±27.6	475.7±17.8	492.6±48.7	465.2±13.6
EF (%)	66.2±5.2	23.8±2.9#	34.8±7.7#	32.9±4.8#
SW (mmHg* µl)	761.8±57.9	310.8±33.6#	354.7±60.6#	413±49.9#
dP/dt max (mmHg/s)	6337.5±593.6	5053.4±343.5	6272.7±631.4	5540.4±321.1
dP/dt min (mmHg/s)	−5961.9±629.6	−3961.7±286.3#	−4807.3±388.4	−4701.1±297.2
SV ( µl)	11.9±0.9	6.8±0.6#	6.5±1#	7.5±0.9#
CO ( µl/min)	5245.9±417.4	3231±307#	3346±711.5#	3541.6±514.5#
Ves ( µl)	7.8±1	25.7±2.5#	16.1±3.3	19.2±2.9^#^
Ved ( µl)	18.7±1.3	31.2±2.2#	21.6±3.8	25.3±3.1

Values are means ± SEM. HR, heart rate; EF, ejection fraction; SW, stroke work; dP/dt max, maximum first derivative of change in pressure rise with respect to time; dP/dt min, maximum first derivative of change in pressure fall with respect to time; SV, Stroke volume; CO, cardiac output; Ves, end-systolic volume; Ved, end-diastolic volume; One-way ANOVA post hoc Tukey's Multiple Comparison Test # p<0.05 vs. sham; no significant differences vs. control MI.

**Figure 3:**
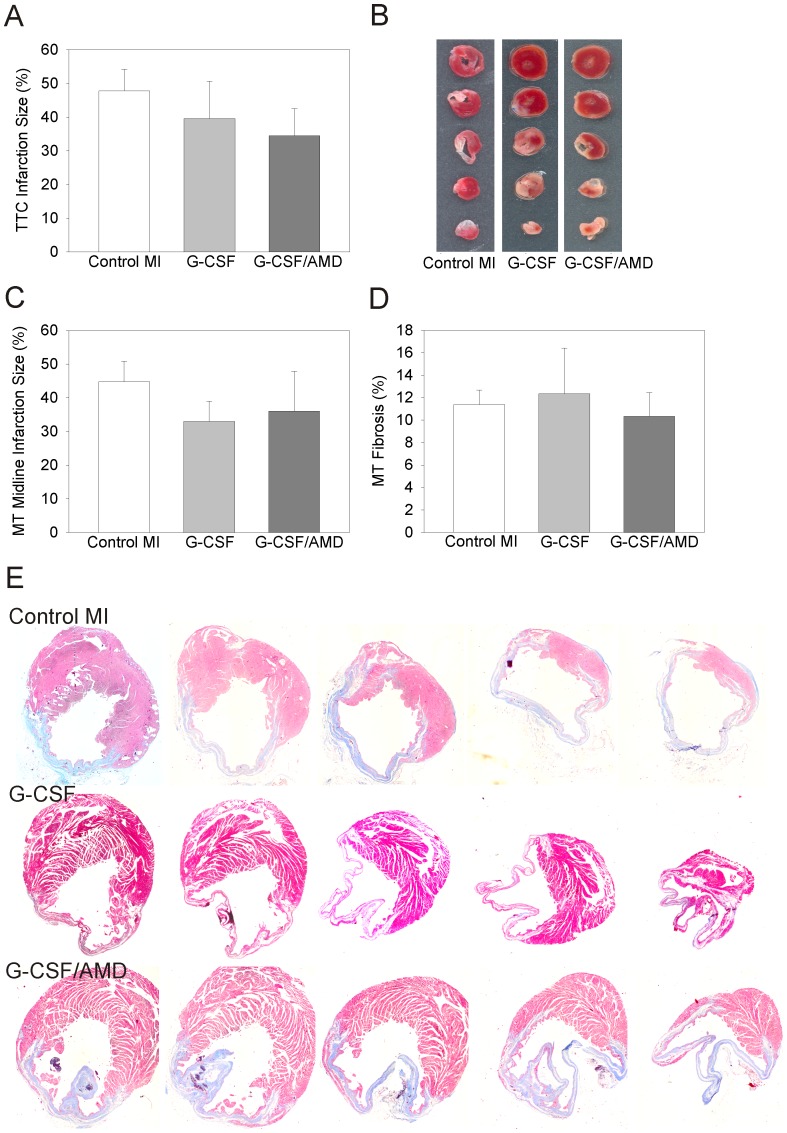
TTC and Masson trichrome staining of infarcted cardiac tissue for assessment of infarction size and fibrosis. Infarction size expressed as percentage of left ventricular area of control MI and drug treated mice assessed by TTC (**A, B**) and Masson trichrome (**C**) staining 28 days after MI. (**D, E**) Masson trichrome staining of sequential heart sections of control MI, G-CSF and G-CSF/AMD treated mice reveals no difference in left ventricular dilation, infarction size and fibrosis.

Determination of infarction size 28 days post MI either by TTC staining or midline infarct length method on Masson trichrome stained sections revealed no significant difference between drug treated animals and control MI mice (Figure 3 A, B, C). Quantification of collagen rich fibrotic areas of the infarcted hearts on sequential transversal sections of Masson trichrome stained sections revealed no differences in fibrosis between control MI mice and drug treated groups (Figure 3 D, E). In summary, neither single G-CSF nor combinatorial G-CSF/AMD therapy significantly altered left ventricular hemodynamics and infarction size.

### Cardiac histology and vascularization

**Figure 4:**
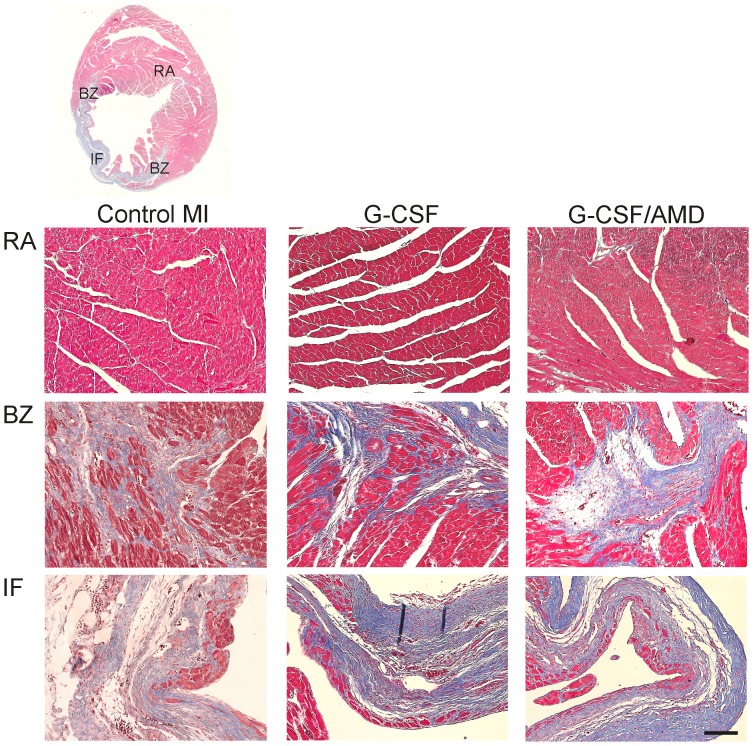
Cardiac histology of infarcted hearts 28 days after MI. Overview of Masson trichrome stained heart section **(upper panel)** and higher magnification images **(lower panels)** of border zone (BZ), infarcted region (IF) and remote area (RA). Images show no evident alteration of collagen deposition in designated areas between treatment groups. Bar: 100 µm.

Twenty-eight days after LAD ligation characteristic signs of late phase postinfarction remodelling of the heart were visible. Infarcted left ventricles showed typical loss of myocardium, left ventricular wall thinning and fibrous scar formation. Masson trichrome staining of infarcted regions revealed a viable subendocardial layer followed by collagen rich fibrous tissue reaching into border zone myocardium (Figure 4). No obvious differences in quantity of viable myocardium as well as interstitial collagen deposition in border zone or remote areas between control MI and drug treated mice hearts were visible.

**Figure 5:**
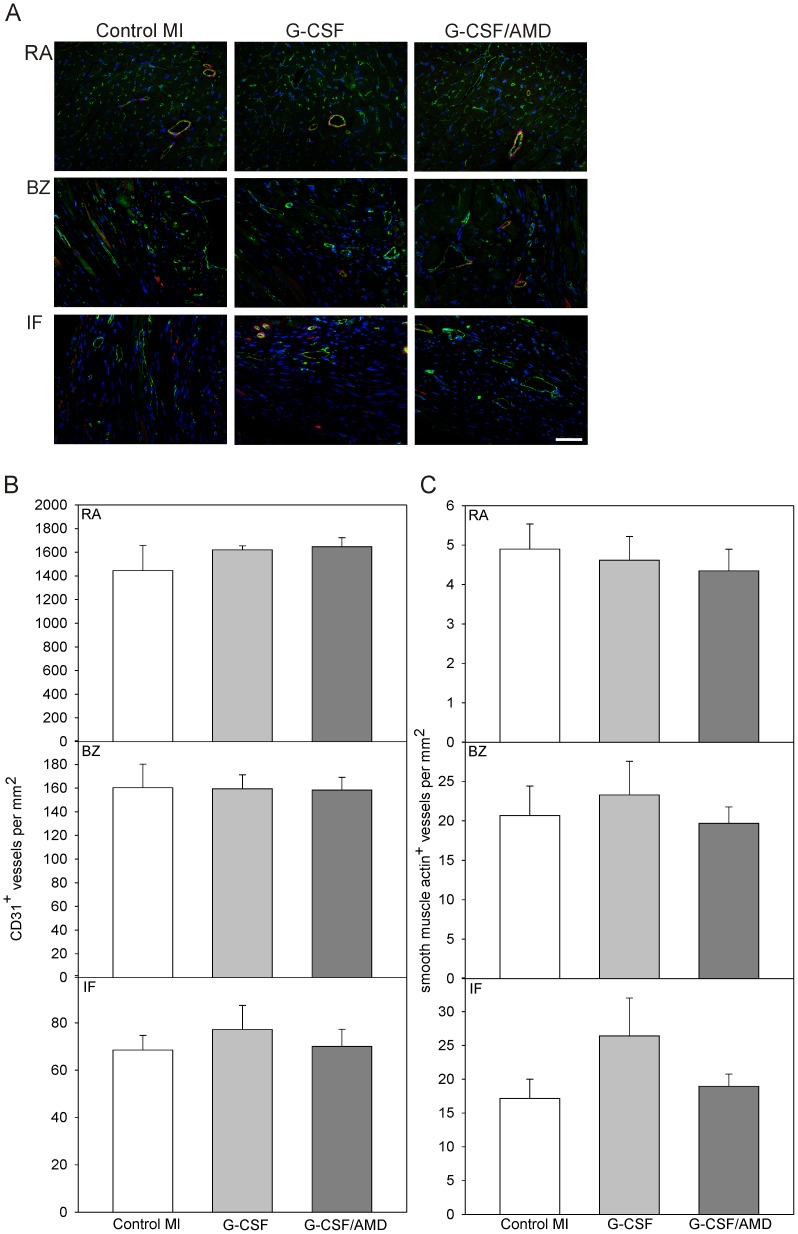
Cardiac vascularization 28 days after MI. Blood vessel formation was analyzed by immunfluorescence staining with specific antibodies against endothelial (CD31/PECAM-1) and smooth muscle (α-smooth muscle actin) cells (**A**). Comparable density of (**B**) CD31/PECAM-1 and (**C**) smooth muscle actin positive vascular structures in the remote area (RA, upper panel), border zone (BZ, middle panel) and infarcted area (IF, lower panel) among control MI and drug-treated groups. Data represent means ± SEM. (n = 5−8 hearts per group).

EPC as well as certain subsets of WBC are known to exert angiogenic properties in ischemic tissues. Using antibodies against endothelial (CD31/PECAM-1) and smooth muscle cells (α-smooth muscle actin), the abundance of capillaries and arterioles in remote area (RA), border zone (BZ) and infarct zone (IF) was analyzed (Figure 5 A). Treatment with G-CSF and G-CSF/AMD had no significant effect on the vessel density in any of the designated regions compared to the control MI group (see Figure 5 B, C).

## Discussion

In the present study, the potential regenerative properties of a combinatorial G-CSF/AMD therapy were tested in a model of permanent LAD occlusion. During the last decade numerous studies investigated the hypothesis that cytokine mediated mobilization of stem cells contribute to cardiac regeneration after MI. Implementing different cytokines and mobilization protocols these studies yielded controversial results ranging from beneficial effects to even deleterious effects on cardiac regeneration [20], [21], [22], [23], [24], [25], [26], [27], [28]. Moreover, the precise mode of action of stem cells in cardiac repair is still a matter of debate. While some studies showed direct differentiation of stem cells into functional cardiomyocytes [29], [30], [31], these results could not be reproduced by others [32], [33] supposing paracrine effects on surrounding cells to be the cause of regeneration in ischemic tissues [34], [35]. In addition, a number of studies proposed that the applied cytokine itself directly influences survival of myocytes and endothelial cells [22], [23], [24], [25], [26], [36], thereby promoting myocardial recovery and neovascularization. In view of the fact that cardiac regeneration certainly involves interplay of complex protective mechanisms this study was aimed to optimize the mobilization of progenitor cells and combine it with possible cytoprotective effects of the most widely used mobilizing cytokine G-CSF. Therefore, we implemented a mobilization scheme in which G-CSF was applied in a relatively high dosage (250 µg/kg) for a short period of 3 days starting directly after induction of MI. This experimental setting was directed to support early cytoprotective actions [5] while avoiding long-term detrimental effects of G-CSF promoted inflammatory processes [20], [37].

G-CSF as well as the CXCR4 inhibitor AMD3100 has been shown to mobilize HSPC and potential angiogenic cells from BM [38], [39], [40]. However, both agents exhibit different mobilization kinetics. While G-CSF leads to a delayed mobilization [41], [42], AMD was shown to be a rapid mobilizer leading to a peak mobilization after 1–3 hours in mice [8], [41]. Combination of both has been proven to synergistically enhance HSPC mobilization in mice [8] and humans [10] with the potential to promote neovascularization in a mouse model of hindlimb ischemia [11]. Although considered as a reliable mobilizing agent, continuous AMD application seems to attenuate positive effects of stem cell mobilization due to blockade of SDF-1α/CXCR4 mediated stem cell homing [13], [14]. In view of these results, we combined G-CSF therapy with a single AMD administration at day 3 after MI to further enhance G-CSF mediated stem cell mobilization while avoiding negative effects of long term AMD application on stem cell homing.

In the present study we found that G-CSF and G-CSF/AMD treatment promoted mobilization of WBC and EPC into peripheral blood. Mobilization tended to be higher in treatment groups compared to control mice without reaching statistical significance. This was due to large variations within treatment groups suggesting that not all animals responded to the treatment in the same way. Inter-individual variations in G-CSF induced HSPC mobilization are also evident in humans [43]. A clear mobilization of CD34^+^ cells, but with huge animal-to-animal variations was also seen in a rat model of MI after G-CSF treatment by Werneck-de-Castro et al. [27]. Nevertheless, these inter-individual variations in stem cell mobilization reflect the practical circumstances that a regenerative therapy has to face.

Combination of G-CSF with AMD did not synergistically increase WBC counts, HPC or EPC numbers at day 7 after MI (4 days after AMD treatment). The rapid AMD mediated mobilization might be an explanation for this, however, significantly increased EPC mobilization could be detected even 7 days after single AMD injection in a mouse MI model [12]. Furthermore, the rather moderate mobilization in the present study could be due to the specification of HPC and EPC as c-Kit/Sca-1 or Flk-1/Sca-1 double positive cells that likely defines a more specific subtype than often used single CD34^+^ or CD133^+^ HSPC.

Survival analysis showed no significant differences in mortality between saline and drug treated groups. On the basis of the applied drug regimen, beneficial effects on cardiac recovery resulting from either direct or paracrine actions of mobilized stem cells would not be conceivable until day 4 after MI. However, exclusion of animals that died in the first 4 days after MI did not uncover a reduced mortality of drug treated animals. Furthermore, pressure volume relationships of control and drug treated animals were recorded to evaluate heart function 28 days after MI. Drastically reduced heart function was evident in control MI mice compared to sham operated mice. Although G-CSF and G-CSF/AMD treatment led to a slight improvement of some hemodynamic parameters, no significant changes compared to the control MI group were observable. Moreover, there was no reduction in infarction size visible in drug-treated versus control MI animals. Since these basic parameters indicated no improvement of heart regeneration with respect to the applied therapy, histology and vascularization of infarcted hearts was evaluated. Histological analysis showed drastically reduced myocardium at the ischemic site of the left ventricle that was replaced by a thin collagen rich, fibrous tissue layer. In control MI as well as treatment groups similar histological pattern were visible showing no obvious signs of cardiac regeneration.

Besides postulated direct actions of cytokines or stem cells on myocyte regeneration, numerous studies linked HSPC mobilization to favorable angiogenic effects promoting neovascularization of ischemic tissues [11], [14], [25], [44], [45]. On that account, formation of capillaries and arterioles was determined in the remote area, border zone and infarcted area of control MI and drug treated animals 28 days after MI. There were no indications for significantly altered vascularization in any region of the heart among MI groups. These results indicated that the applied drug regimen did not provoke measurable vasculogenic properties. This is in conflict with studies showing G-CSF and AMD induced neovascularization [11], [12], [45]. However, absent effects on cardiac vascularization [20] and even inhibitory actions of G-CSF on vascular tubule formation and vascularization of subcutaneous sponges [46] have been reported by others. The inflammatory response after myocardial ischemia plays a pivotal role in heart regeneration being accountable for positive as well as adverse outcomes [47], [48], [49]. Elevated WBC numbers, reported in this study and by others [11], [20], [21] are capable to induce increased inflammation and adverse events after myocardial infarction [37], [50]. In a study of Maekawa et al. [51] induction of the closely related cytokine granulocyte-macrophage stimulating factor (GM-CSF) led to increased macrophage infiltration into the infarcted myocardium. Moreover, expression of collagen and fibrogenic TGF-β1 was increased 14 days after MI. These effects resulted in infarct expansion, aggravated cardiac remodeling and increased mortality of treated rats after permanent LAD ligation. Cheng et al. [20] reported that G-CSF therapy affects expression of matrix-metalloproteinases (MMP) and their tissue inhibitors (TIMP) leading to increased fibrosis, mortality and left ventricular dysfunction after MI in the long term. Our implemented treatment regimen with G-CSF and G-CSF/AMD did not provoke negative effects on myocardial regeneration. This might be due to the short time period of drug therapy starting directly after MI. Numerous studies showed beneficial effects of mesenchymal stromal cell (MSC)- infusion on MI recovery in rodents and humans [52]. In a study of Pitchford et al. combinatorial G-CSF/AMD3100 treatment resulted in elevated peripheral EPC and HSC but not stromal progenitor cells (SPC) [53]. Furthermore single G-CSF therapy in patients after percutaneous intervention surprisingly led to decreased numbers of putative MSC in peripheral blood and had no effect on systolic performance [54]. From these observations it is tempting to speculate that missing MSC mobilization might be responsible for the poor regenerative properties of G-CSF or G-CSF/AMD therapy. However, the precise actions of G-CSF and cell based therapies in cardiac regeneration are uncertain and divergent results ranging from multi-level benefits to adverse effects on cardiac remodeling could be observed. It must be carefully taken into account that in addition to differences in experimental design, animal dependent factors such as genetic background, age, body temperature, and even colony substrain differences critically influence the susceptibility to myocardial ischemia and cardiac healing [55], [56].

In conclusion, although the applied drug regimen enhanced the mobilization of potentially regenerative cells, the combination of G-CSF and AMD did not significantly improve cardiac recovery after MI compared to control MI mice. On the other hand, no adverse effects of the applied drug treatment on cardiac function and remodeling could be observed. Further studies are needed to elucidate the complex mechanisms after cardiac injury to figure out treatment regimens that more specifically promote cardiac healing.

### Study limitations

Beneficial effects of stem cells or cytokines were already observed after permanent LAD occlusion [25], [57] however, this frequently used model has the limitation that it does not reflect the actual conditions in patients after angioplasty. The type of model (permanent vs. transient LAD occlusion) could influence the regenerative capacities of G-CSF therapy as suggested for experimental stroke models [58].

## Supporting Information

Figure S1 **FACS analysis of peripheral white blood cells of naïve (day 0) mice and 7 days post MI.** (A) Mononuclear cells were gated on forward scatter (FSC) and side scatter (SSC) plot to exclude blood cells, debris and dead cells. Percentages of (B) c-Kit/Sca-1 double positive and (C) Flk1/Sca-1 double positive sub-populations were recorded. Representative dots plots are shown.
(TIF)
