# Supplementary figures and images for: Combinatorial G-CSF/AMD3100 Treatment in Cardiac Repair after Myocardial Infarction
Source: PLoS One. 2014 Aug 14;9(8):e104644. doi: 10.1371/journal.pone.0104644 (PMC4133256; doi:10.1371/journal.pone.0104644)

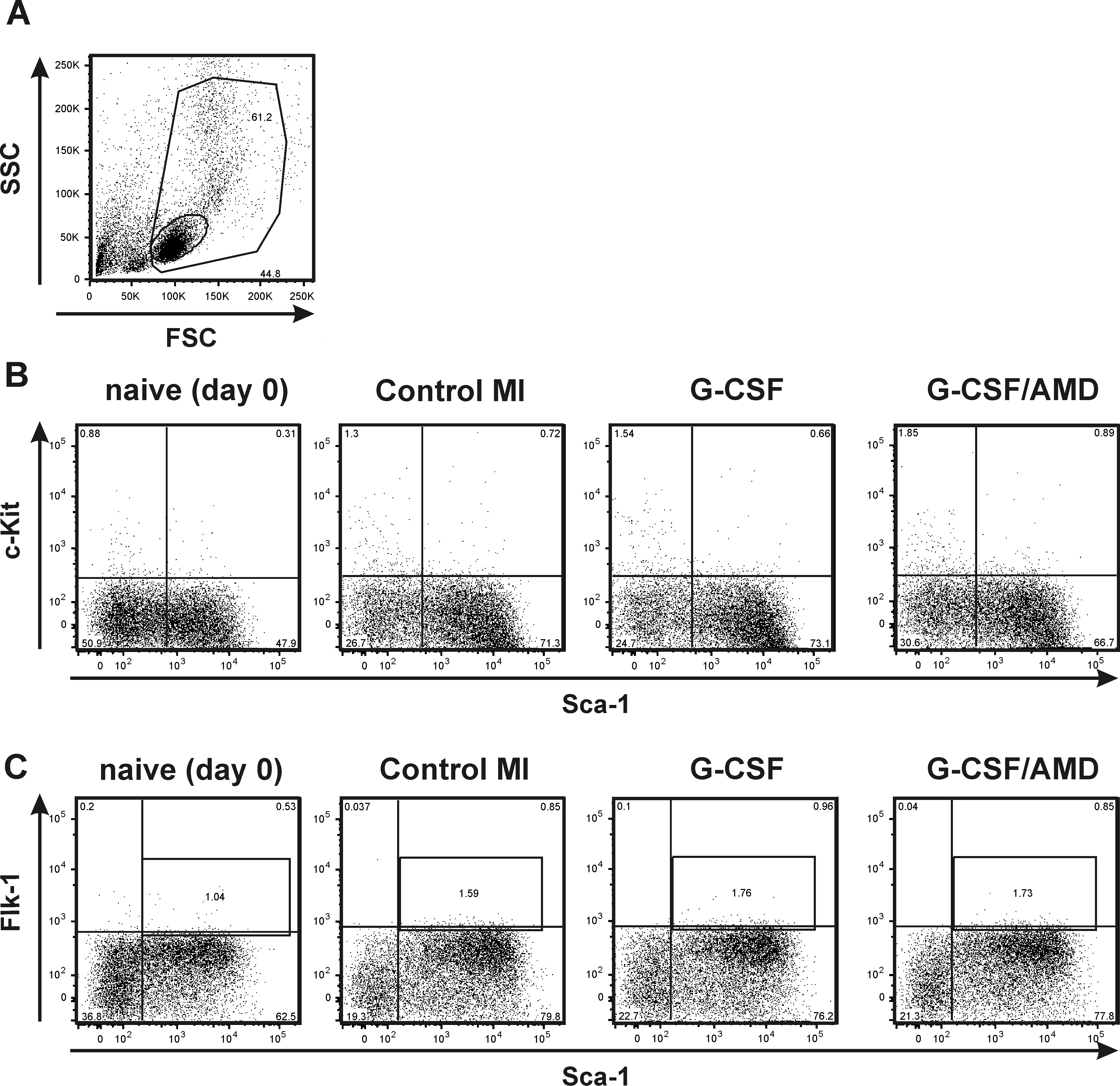

Supplement: Figure S1 — FACS analysis of peripheral white blood cells of naïve (day 0) mice and 7 days post MI. (A) Mononuclear cells were gated on forward scatter (FSC) and side scatter (SSC) plot to exclude blood cells, debris and dead cells. Percentages of (B) c-Kit/Sca-1 double positive and (C) Flk1/Sca-1 double positive sub-populations were recorded. Representative dots plots are shown. (TIF) [file pone.0104644.s001.tif]
